# Dengue-specific T-cell memory following natural infection: a comparative analysis between people living with HIV and HIV-negative individuals

**DOI:** 10.1128/spectrum.02899-25

**Published:** 2026-05-07

**Authors:** Francesca Lombardi, Antonio Abatino, Pierluigi Francesco Salvo, Ilenia Aversa, Gianmaria Baldin, Raffaella Gallo, Enrica Tamburrini, Patrizia Laurenti, Valeria Campolattano, Simona Di Giambenedetto, Carlo Torti, Camillo Palmieri

**Affiliations:** 1 Dipartimento di Sicurezza e Bioetica, Sezione Malattie Infettive, Università Cattolica del Sacro Cuore 96983 , Rome, Italy; 2 Dipartimento di Scienze Mediche e Chirurgiche, UOC Malattie Infettive, Fondazione Policlinico Universitario A.Gemelli IRCCS 18654, Rome, Italy; 3 Department of Experimental and Clinical Medicine, University of Catanzaro "Magna Graecia" 9325 , Catanzaro, Italy; 4 Department of Life Sciences and Public Health, Università Cattolica del Sacro Cuore 96983 , Rome, Italy; 5 Department of Women, Child and Public Health Sciences, Fondazione Policlinico Universitario A.Gemelli IRCCS 18654, Rome, Italy; Barnard College, Columbia University, New York, New York, USA

**Keywords:** HIV-1, dengue infection, dengue vaccination

## Abstract

People living with HIV (PLWH) represent a population of particular interest in the context of arboviral infections. This observational study aimed to evaluate and compare the magnitude and functionality of dengue virus (DENV)-specific T-cell memory elicited by natural DENV infection in PLWH and HIV-negative individuals. Nineteen participants infected during the 2023 autochthonous dengue outbreak in Rome (10 PLWH and 9 HIV-negative individuals) were assessed for interferon gamma (IFN-γ)-releasing cells in peripheral blood mononuclear cells (PBMCs) using an IFN-γ -ELISpot assay following stimulation with peptide pools.When examining both the overall cumulative ELISpot responses, calculated as the sum of spot-forming units (SFU) across all antigens or the binary responder status to each DENV antigen, HIV-negative individuals showed higher and broader cumulative responses than PLWH, but differences did not reach statistical significance. However, responses to prM/E/C antigens were higher in the HIV-negative group, reaching statistical significance (*P* = 0.041). Among PLWH, no association was found between T-cell reactivity and CDC stage at diagnosis. Our findings suggest that while PLWH with well-controlled infection retain dengue-specific T-cell memory, their response to structural antigens may be attenuated. Vaccination should be considered a key preventive strategy for this clinically vulnerable population.

**IMPORTANCE:**

The geographic expansion of dengue virus (DENV) transmission into temperate regions highlights the need to characterize immune responses in populations with chronic conditions such as HIV. Despite immune reconstitution under antiretroviral therapy (ART), people living with HIV (PLWH) may exhibit residual immune dysregulation that could affect DENV-specific immunity and clinical outcomes. In this study, we directly compared DENV-specific T-cell memory responses in PLWH and HIV-negative individuals following natural infection during an autochthonous outbreak. While PLWH mounted detectable cellular responses, reactivity to structural antigens (prM/E/C) was significantly reduced compared with HIV-negative participants. These findings indicate that qualitative differences in antigen-specific T-cell memory may persist despite virological suppression and immune recovery, with potential implications for protection upon re-exposure. Our results provide a rationale for evaluating dengue vaccination strategies in PLWH as local transmission becomes more frequent in non-endemic settings.

## INTRODUCTION

Dengue virus (DENV) infection represents a major global health challenge, with an estimated 390 million infections occurring annually and approximately 3.9 billion people living in areas at risk of transmission (1). Although traditionally confined to tropical and subtropical regions, the geographic distribution of dengue has expanded significantly over the past decades due to factors such as urbanization, global travel, climate change, and the spread of competent mosquito vectors (*Aedes aegypti* and *Aedes albopictus*) (2). This expansion has led to the emergence of autochthonous dengue outbreaks in previously non-endemic areas, including parts of Southern Europe, as exemplified by the outbreak reported in Rome, Italy, in 2023, which underscores the potential for local transmission in temperate climate regions and highlights the need for improved surveillance and a deeper understanding of dengue immunopathogenesis in diverse populations (3).

People living with HIV (PLWH) represent a population of particular interest in the context of arboviral infections. While antiretroviral therapy (ART) has markedly improved life expectancy and immune function in PLWH, persistent immune dysregulation and inflammation may affect their response to coinfections, including dengue (4, 5). Observational studies (6, 7) and case reports (8, 9) suggest that HIV infection may influence both the clinical presentation and the outcomes of dengue, with some evidence indicating an increased risk of progression to severe forms of the disease, particularly in the context of secondary dengue infection and/or suboptimal immune recovery under ART (5, 8).

T-cell-mediated immunity plays an important role in the host response to DENV (10, 11). CD4 and CD8 T cells contribute to viral clearance with the production of proinflammatory cytokines, cytotoxic activity, and the establishment of long-term immunological memory. However, T-cell responses may also contribute to immunopathology, particularly in the setting of heterologous secondary infections (11, 12). In PLWH, HIV infection may impair both the magnitude and the quality of antigen-specific T-cell responses, potentially altering the balance between protective and pathogenic immunity following DENV infection (13).

Although the impact of HIV on T-cell function suggests that DENV-specific cellular immunity may be altered in PLWH, the topic remains unexplored.

This study aimed to evaluate and compare the magnitude and functionality of DENV-specific T-cell memory elicited by previous natural DENV infection in PLWH and HIV-negative individuals.

## MATERIALS AND METHODS

### Study design

An observational study was performed on 19 individuals who acquired autochthonous dengue virus infection during the outbreak reported in Rome in 2023 (14). The study population included 10 PLWH and 9 HIV-negative participants.

To compare the magnitude and functionality of DENV-specific T-cell memory elicited by natural DENV infection, we measured the frequency of interferon gamma (IFN-γ)-releasing cells in peripheral blood mononuclear cells (PBMCs) using an IFN γ -ELISpot assay, following stimulation with peptide pools. Peptides were designed to represent all four DENV serotypes and to cover the most prevalent HLA class I and class II restrictions (Table S1). A T-cell response was considered positive when IFN-γ production was detected in response to at least one of the tested antigen pools.

### PBMC purification

Peripheral venous blood was collected in EDTA vacutainer tubes, and PBMCs were isolated by density gradient isolation using Ficoll-Paque (Merck, KGaA, Darmstadt, Germany), according to the manufacturer’s instructions. The isolated PBMCs were immediately cryopreserved and stored in liquid nitrogen until use.

### Enzyme-linked immunoSpot assay

Enzyme-linked immunoSpot (ELISpot) was used for the enumeration of PBMCs secreting interferon gamma (IFN-γ) in response to peptide pools derived from both structural (prM, E, and C) and nonstructural (NS2A/B, NS3, NS4A/B, and NS5) DENV antigens (listed in Table S1), as previously described (15, 16). Briefly, the cryopreserved PBMCs were thawed and left to rest overnight in complete activation-induced cell marker medium (ThermoFisher Scientific, Waltham, MA, USA). Then, ELISpot plates precoated with human IFN-γ antibody (mAb1-D1K) were seeded with 400,000 PBMCs/well and stimulated for 18 h with 2 μg/mL of the peptide pool, anti-CD3/CD28 (0.1 μg/mL; positive control), or dimethyl sulfoxide (DMSO, negative control). Then, wells were washed five times with PBS containing 0.05% Tween-20 and incubated for 2 h at room temperature with 100 µL per well of a biotinylated anti–IFN-γ detection antibody (mAb7-B6-1, 0.2 µg/mL in PBS-Tween). After another five washes, plates were incubated for 1  h with streptavidin–alkaline phosphatase (1:1,000 dilution) to reveal bound biotin. Following a final series of washes, spots were developed by adding 100 µL per well of BCIP/NBT-plus substrate and incubating in the dark for 5–10 min, until distinct purple spots appeared. The reaction was stopped by rinsing plates under running tap water, and membranes were allowed to air-dry. Spots corresponding to stimulated cells secreting IFN-γ were counted by an immunoSpot plate analyzer (BIOREADER3000; Bio-Sys, Germany). The IFN-γ-ELISpot data were reported as stimulating forming units × 10^6^ PBMCs (SFU/10^6^), which was calculated for each PBMC sample by subtracting spots of the unstimulated wells from the spots of the peptide-stimulated wells and normalizing to 10^6^ PBMCs (15, 16). Results were expressed as individual DENV-specific or cumulative IFN**-γ** -ELISpot responses. Cumulative ELISpot responses were calculated as the sum of SFUs across all peptide pools or for each antigen within the group.

### Statistical analysis

Statistical analyses were performed with GraphPad PRISM software 9.3 (GraphPad Software, La Jolla, CA, USA). Statistical tests were selected based on appropriate assumptions with respect to data distribution and variance characteristics; *P* < 0.05 was considered statistically significant. Proportions were compared using χ2 or Fisher’s exact test, as appropriate. Statistical comparisons between groups for each antigen were performed using the nonparametric two‐sided Mann-Whitney *U* tests.

## RESULTS

Our cohort included 10 PLWH with a median age of 56 years (IQR 43–60) and 9 HIV-negative individuals with a median age of 42 years (IQR 39–67). Table 1 presents clinical and demographic characteristics for each participant. Sex distribution was balanced in both groups, with five females and five males among PLWH and four females and five males in the HIV-negative group. The interval between DENV infection and sample collection was similar in both groups, with a median of 8.8 months since infection in PLWH and 9.7 months in HIV-negative individuals. Among PLWH, according to the CDC classification, four PLWH were in category A (asymptomatic), four in category C (AIDS-defining conditions), and two in category B (symptomatic but not AIDS-defining).

**TABLE 1:** Clinical and immunological characteristics of study participants

ID	Sex	Age (years)	HIV status	CDC^*a*^	Time since HIV diagnosis (years)	Time on ART^*b*^ (years)	ART^*b*^	CD4+ T cells/mL	CD8+ T cells/mL	CD4/CD8	HIV-RNA	Time from DENV infection (months)	SFU/10^6^ PBMCs^*c*^
PT1	F	51	POS	C	15.5	15.4	3TC/TDF/ DOR	684	528	1.3	TND	8.8	7
PT2	M	56	POS	C	12.0	12.0	3TC/DTG	515	611	0.84	TND	6.3	3
PT3	M	57	POS	C	5.8	5.7	3TC/TDF/ DOR	427	1085	0.39	TND	8.9	12
PT4	F	76	POS	A	28.6	26.9	3TC/DTG	317	795	0.4	TND	9.1	5
PT5	M	30	POS	A	7.9	7.8	3TC/DTG	949	758	1.25	TND	8.4	3
PT6	F	67	POS	B	18.1	17.9	3TC/DTG	511	546	0.94	TND	7.5	44
PT7	M	37	POS	A	5.1	5.1	CAB/RPV	529	532	0.99	TND	10.3	12
PT8	F	55	POS	C	23.7	22.6	TAF/FTC/ BIC	657	472	1.39	TND	10.8	2
PT9	M	41	POS	B	5.7	5.6	3TC/DTG	540	724	0.75	TND	8.6	6
PT10	M	61	POS	A	29.1	24.7	TAF/FTC/ RPV	816	1189	0.69	TND	4.8	14
PT11	F	42	NEG	NA	NA	NA	NA	NA	NA	NA	NA	9.7	113
PT12	F	38	NEG	NA	NA	NA	NA	NA	NA	NA	NA	10.1	167
PT13	M	80	NEG	NA	NA	NA	NA	NA	NA	NA	NA	9.2	46
PT14	M	28	NEG	NA	NA	NA	NA	NA	NA	NA	NA	8.8	7
PT15	M	40	NEG	NA	NA	NA	NA	NA	NA	NA	NA	9.4	0
PT16	F	22	NEG	NA	NA	NA	NA	NA	NA	NA	NA	7.6	12
PT17	M	78	NEG	NA	NA	NA	NA	NA	NA	NA	NA	9.1	12
PT18	M	67	NEG	NA	NA	NA	NA	NA	NA	NA	NA	10.8	16
PT19	F	58	NEG	NA	NA	NA	NA	NA	NA	NA	NA	10.5	6

^*a*^ HIV disease stage according to CDC classification at the time of HIV diagnosis.

^*b*^ ART, antiretroviral therapy, regimen at the time of sample collection; 3TC, lamivudine; TDF, tenofovir disoproxil fumarato; DOR, doravirine; DTG, dolutegravir; CAB, cabotegravir; RPV, rilpivirine; TAF, tenofovir alafenamide fumarato; FTC, emtricitabine; BIC, bictegravir; TND, target nondetected (HIV-RNA 0 copy/mL); NA, not applicable.

^*c*^ Cumulative spot-forming units per million peripheral blood mononuclear cells (PBMCs), as measured by ELISpot assay.

All PLWH (10/10) and all but one HIV-negative participant (8/9) exhibited a positive T-cell response to at least one of the stimulating peptide pools.

**Fig 1:**
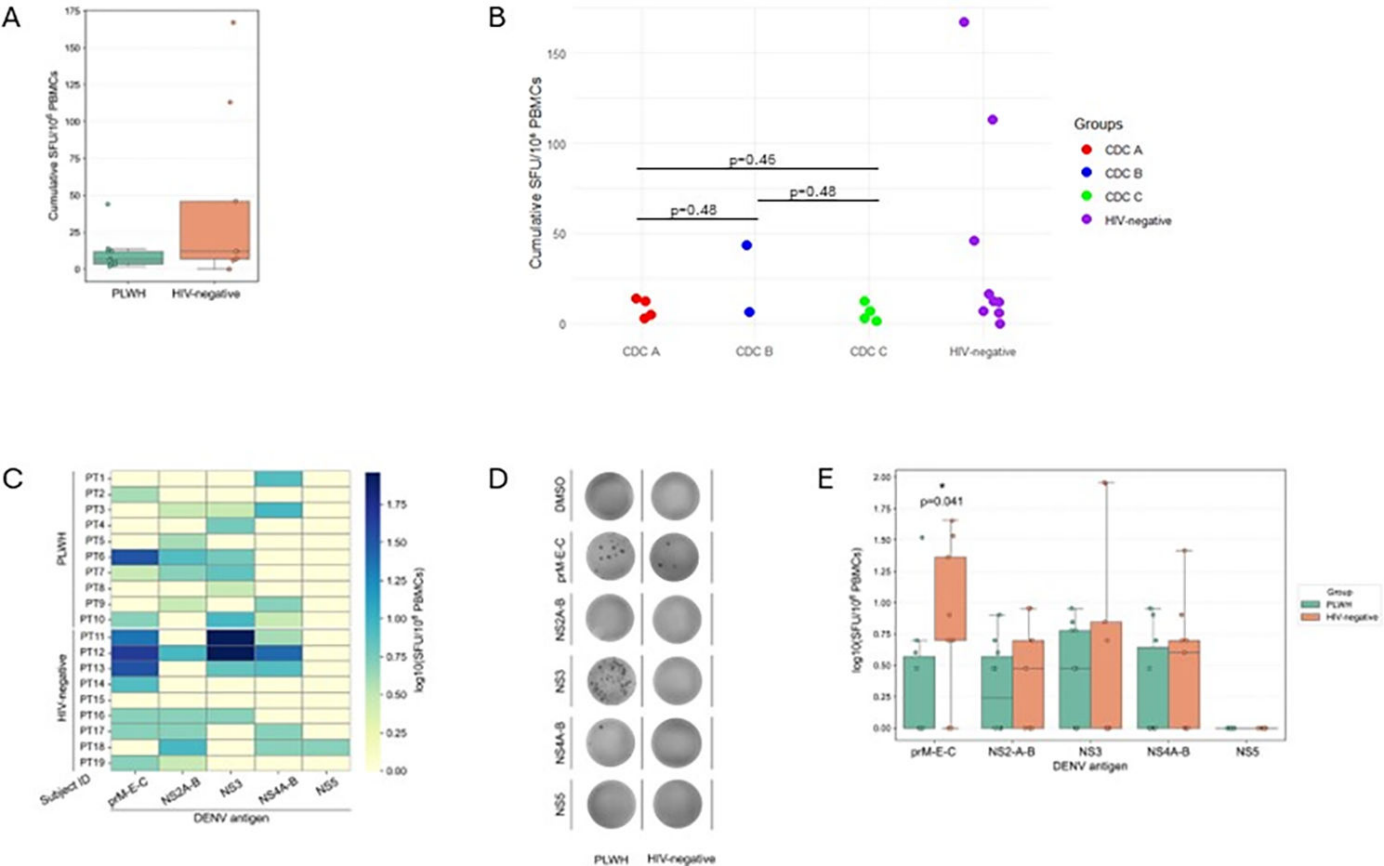
DENV-specific IFN-γ ELISpot responses in PLWH and HIV-negative individuals. (**A**) Boxplot of cumulative IFN-γ ELISpot responses. Cumulative responses were calculated as the sum of SFUs across all peptide pools for each donor PLWH (*n* = 10) and HIV-negative (*n* = 9) are shown as individual points overlaid on the box-and-whisker plots (boxes = IQR, horizontal line = median, and whiskers = 5th–95th percentiles). (**B**) DotPlot of individual DENV-specific IFN-**γ** ELISpot responses for HIV disease stage according to CDC classification at the time of HIV diagnosis among PLWH and HIV-negative participants. (**C**) Heatmap summary of individual DENV-specific IFN**-γ** -ELISpot responses for PLWH and HIV-negative participants. (**D**) Representative of individual DENV-specific IFN**-γ** -ELISpot responses of individual samples from PLWH (PT2) and HIV-negative (PT11) group. Each sample was left unstimulated (DMSO) or stimulated with the indicated DENV-peptide pool. (**E**) Boxplot of DENV antigen-specific IFN-γ ELISpot responses.

Cumulative ELISpot responses across all antigens were slightly higher in HIV-negative individuals (median: 12.0; IQR: 7–46) compared to PLWH (median: 5.0; IQR: 3–12); however, this difference did not reach statistical significance (Mann-Whitney *U*, *P*  =  0.201). (Fig. 1A).

Among PLWH, no significant differences were observed between CDC stage A (median = 8.5 SFU) and stage B (median = 25 SFU) (*P* = 0.48, Mann-Whitney *U* test); between CDC stage A and stage C (median = 5 SFU) (*P* = 0.46, Mann-Whitney *U* test); and between CDC stage B and stage C (*P* = 0.48, Mann-Whitney *U* test) (Fig. 1B).

Analysis of binary responder status to each DENV antigen pool showed that HIV-negative individuals tended to recognize a broader range of antigens, although none of the differences reached statistical significance (Fig. 1C). Responder frequencies for prM/E/C were 7/9 (77.8%) in HIV-negative versus 4/10 (40.0%) in PLWH (Fisher exact test, *P* = 0.17); for NS2A/B, 5/9 (55.6%) versus 5/10 (50.0%) (*P* = 1.00); for NS3, 4/9 (44.4%) versus 6/10 (60.0%) (*P* = 0.66); and for NS4A/B, 5/9 (55.6%) versus 4/10 (40.0%) (*P* = 0.66). A single response to NS5[FL4.1] was detected in one out of nine HIV-negative individuals (11.1%), while no responses were observed in the HIV-positive group (0/10; *P* = 1.00)

Regarding the response magnitude, the highest ELISpot values were observed for the NS3 antigen, but these were limited to two HIV-negative individuals (PT11 and PT12), who showed markedly elevated responses (88 and 90 SFU) (Fig. 1D).

Median NS3 response remained low in both groups, and this skewed distribution in the HIV-negative cohort likely reflects individual variability in T-cell memory rather than a consistent group-level difference (Fig. 1E). No statistically significant difference was found between groups in NS3-specific responses (*P* = 1.000, Mann-Whitney *U* test). In contrast, responses to prM/E/C were more evenly distributed and higher in the HIV-negative group (median: 22.0 SFU) compared to PLWH (median: 2.5 SFU), reaching statistical significance (*P* = 0.041).

## DISCUSSION

This study aimed to assess whether PLWH with well-controlled infection can develop and maintain functional DENV-specific T-cell memory after natural infection. This question is particularly relevant because PLWH represent a clinically vulnerable population in the context of secondary dengue infection due to both viro-immunological characteristics and the immunopathogenic nature of heterologous re-infection. Despite this risk, the cellular immune responses to DENV in PLWH remain poorly characterized. Existing literature has primarily focused on the clinical presentation and outcomes, with only a few case reports or observational studies addressing T-cell immunity in this population (5, 7, 8), leaving a substantial gap in our understanding of antigen-specific T-cell memory following DENV infection in PLWH. To our knowledge, this is the first study to directly compare DENV-specific T-cell memory responses between PLWH and HIV-negative individuals using IFN-γ ELISpot assays.

In our cohort, all PLWH—who were virologically suppressed and had CD4+ counts > 400 cells/µL—developed detectable DENV-specific T-cell responses. When examining both the overall cumulative ELISpot responses, calculated as the sum of spot-forming units across all antigens or the binary responder status to each DENV antigen, HIV-negative individuals showed higher and broader cumulative responses than PLWH, although differences did not reach statistical significance (Fig. 1A). Notably, cumulative responses were similar between PLWH classified as CDC stage A and CDC stage C at diagnosis, suggesting that the past disease stage does not significantly influence the ability to develop DENV-specific T-cell memory under ART. However, despite preserved immune control, PLWH showed significantly lower responses to structural antigens (prM/E/C), which are key targets of protective immunity. This attenuation may reflect subtle immune dysregulation persisting despite ART and could indicate a less robust induction of antigen-specific T-cell response. These findings support the rationale for considering dengue vaccination in well-controlled PLWH, especially in regions where the risk of re-exposure is increasing.

In agreement with our data, several studies showed that immune response to viral infections in PLWH depends on their immune competency. PLWH with well-controlled HIV infection show a humoral and T-cell response comparable to HIV-uninfected individuals. Conversely, in ART-naïve individuals with low CD4 counts, generation of T-cell responses is suboptimal and of reduced frequency (17). In our study, reduced reactivity to prM/E/C antigens observed in PLWH could reflect HIV-associated impairments in CD4^+^ T-cell help, antigen presentation efficiency, or T-cell repertoire diversity. Responses to nonstructural antigens such as NS3 were more variable and did not differ significantly between groups, although two HIV-negative individuals showed robust responses, possibly related to HLA-restricted immunodominance.

In a different context, it has been shown that ART-experienced PLWH with suppressed HIV viral load are able to mount a detectable adaptive immune response to SARS-CoV-2 (18). Alrubayyi A. et al. (19) showed via IFN-γ-ELISpot a wide breadth and range of cumulative SARS-CoV-2 T-cell frequencies in the majority of HIV-positive and -negative donors. However, the proportion of HIV-positive and -negative donors with T-cell responses to individual SARS-CoV-2 pools within given ranges varied, with a higher percentage of HIV-positive donors having low-level responses.

Moreover, in PLWH, the overall magnitude of SARS-CoV-2-specific T-cell responses was related to the size of the naive CD4 T-cell pool and the CD4:CD8 ratio, suggesting that incomplete restoration of the CD4 compartment in ART-treated patients can hinder their ability to mount an optimal T-cell response toward SARS-CoV-2 (19).

Similar patterns of altered T-cell responses in PLWH have been observed in the context of influenza infection. Naturally acquired influenza-specific CD4+ T cell proliferative responses are impaired in PLWH, even in asymptomatic individuals with preserved CD4 counts (> 350 cells/µL). Immune reconstitution following ART initiation is incomplete, with reduced proportions of CD154-expressing influenza-specific CD4+ T cells, suggesting that ART alone may not fully restore antigen-specific T-cell function (20). These data support the concept that HIV-associated immune dysregulation impairs the generation and maintenance of antigen-specific T-cell memory. Our results in the context of DENV infection are therefore consistent with this pattern of attenuated T-cell responses in PLWH, reinforcing the biological plausibility of our findings.

Limitations of this study include the small sample size, which limits generalizability and reduces statistical power to detect subtle differences between groups. The cross-sectional design does not allow assessment of the durability of T-cell memory over time. Additionally, the use of IFN-γ ELISpot assays captures only one functional dimension of T-cell activity, excluding polyfunctional or cytotoxic responses. Of note, the robust responses observed to the anti-CD3/CD28-positive control indicate preserved global T-cell responsiveness, arguing against a state of marked T-cell dysfunction or profound exhaustion in the analyzed samples (Fig. 1D).

Another limitation of this study is that we did not directly assess whether the reduced IFN-γ responses to structural peptides were shared by both memory CD4^+^ and CD8^+^ T-cell subsets. This question was not specifically addressed, mainly due to the limited availability of biological samples, which precluded subset-resolved functional analyses. Nevertheless, the peptides analyzed are of a size compatible with HLA class I presentation (16). While the contribution of other lymphocyte populations cannot be excluded, the data are consistent with the preferential involvement of CD8^+^ T cells. Future studies combining multiparametric flow cytometry with complementary functional assays will be required to comprehensively define the phenotype, activation state, and functional capacity of dengue-specific T cells in PLWH. Lastly, the peptide pools used may not fully represent the diversity of epitopes recognized.

Despite these limitations, our data suggest that PLWH can develop measurable DENV-specific T-cell memory under ART, but their responses to structural viral proteins may be qualitatively less robust. Given the increased risk of severe disease during secondary dengue infection in PLWH, even modest deficits in antigen-specific immunity may have clinical implications.

These findings also raise important considerations for vaccination strategies. Currently, the tetravalent live-attenuated dengue vaccine (TAK-003) is approved in Europe for individuals aged ≥4 years, regardless of prior exposure. However, it is not recommended for immunocompromised individuals, including PLWH, due to limited data on safety and efficacy. Nonetheless, PLWH with asymptomatic infection and CD4+ counts ≥ 200 cells/µL—such as those in our cohort—are generally considered eligible for live-attenuated vaccines without increased risk of adverse events (21). For instance, yellow fever vaccine—another live-attenuated viral vaccine—is currently recommended for PLWH meeting the same immunological criteria, further supporting the potential safety of dengue vaccination in this population.

In conclusion, our findings highlight the ability of PLWH on effective ART to develop DENV-specific T-cell responses, while also revealing attenuated reactivity to structural antigens that may have implications for long-term protection. These results support the need to consider tailored dengue vaccination strategies in this vulnerable population.

## SUPPLEMENTAL MATERIAL

Table S1 spectrum.02899-25-s0001.docx Peptides of DENV proteins used for ELISpot assay.

ASM does not own the copyrights to Supplemental Material that may be linked to, or accessed through, an article. The authors have granted ASM a non-exclusive, world-wide license to publish the Supplemental Material files. Please contact the corresponding author directly for reuse.
