# Supplementary material for: Dengue-specific T-cell memory following natural infection: a comparative analysis between people living with HIV and HIV-negative individuals
Source: Microbiol Spectr. 2026 May 7;14(6):e02899-25. doi: 10.1128/spectrum.02899-25 (PMC13228005; doi:10.1128/spectrum.02899-25)
Supplement: Table S1 — Peptides of DENV proteins used for ELISpot assay. [file spectrum.02899-25-s0001.docx]

**Supplementary table 1. List of peptides of DENV-proteins used for ELISPOT assay**

| **Sequence** | **Protein** | **Start-End** | **Length** |
| --- | --- | --- | --- |
| MLIPTAMAF | C | 107-115 | 9 |
| LPEEQDQNY | E | 362-370 | 9 |
| MSYSMCTGKF | E | 577-586 | 10 |
| SPCKIPFEIM | E | 611-620 | 10 |
| FGAIYGAAF | E | 720-728 | 9 |
| MSFRDLGRVM | NS2A | 1175-1184 | 10 |
| MMATIGIAL | NS2A | 1229-1237 | 9 |
| MAVGMVSIL | NS2B | 1354-1362 | 9 |
| IPMTGPLVAG | NS2B | 1371-1380 | 10 |
| GTSGSPIVDK | NS3 | 1608-1617 | 10 |
| NPEIEDDIF | NS3 | 1650-1659 | 9 |
| HPGAGKTKRY | NS3 | 1670-1679 | 10 |
| APTRVVASEM | NS3 | 1698-1707 | 10 |
| LPIRYQTPAI | NS3 | 1714-1723 | 10 |
| DPASIAARGY | NS3 | 1766-1775 | 10 |
| TPPGSRDPF | NS3 | 1793-1801 | 9 |
| RVIDPRRCLK | NS3 | 1897-1906 | 10 |
| TPEGIIPSM | NS3 | 1976-1984 | 9 |
| GESRKTFVEL | NS3 | 2003-2012 | 10 |
| DLMRRGDLPV | NS3 | 2011-2020 | 10 |
| NYADRRWCF | NS3 | 2032-2040 | 9 |
| RPRWLDART | NS3 | 2068-2076 | 9 |
| MALKDFKEF | NS3 | 2081-2089 | 9 |
| LPTFMTQKAR | NS4A | 2107-2116 | 10 |
| TAEAGGRAY | NS4A | 2127-2135 | 9 |
| TLYAVATTI | NS4B | 2284-2292 | 9 |
| IANQATVLM | NS4B | 2313-2321 | 9 |
| VPLLAIGCY | NS4B | 2336-2344 | 9 |
| FLLVAHYAI | NS4B | 2357-2365 | 9 |
| LILCVTQVLM | NS4B | 2419-2428 | 10 |
| CEALTLATGPISTLW | NS4B | 2436-2450 | 15 |
| SPGKFWNTTI | NS4B | 2453-2462 | 10 |
| IAVSMANIF | NS4B | 2462-2470 | 9 |
| MANIFRGSY | NS4B | 2466-2474 | 9 |
| FTMRHKKATY | NS5 | 2735-2744 | 10 |
| WHYDQDHPY | NS5 | 2784-2792 | 9 |
| WAYHGSYET | NS5 | 2795-2803 | 9 |
| KPWDVIPMV | NS5 | 2822-2830 | 9 |
| TPFGQQRVF | NS5 | 2839-2847 | 9 |
| EPKEGTKKLM | NS5 | 2857-2866 | 10 |
| RPRLCTREEF | NS5 | 2882-2891 | 10 |
| KAKGSRAIW | NS5 | 2959-2967 | 9 |
| KLAEAIFKL | NS5 | 3055-3063 | 9 |
| ETACLGKAY | NS5 | 3242-3250 | 9 |
| MPSMKRFRRE | NS5 | 3375-3384 | 10 |
| HPGFTILALF | prM | 244-253 | 10 |
| MLVTPSMTM | prM | 273-281 | 9 |
